# Stepped-wedge cluster randomised controlled trials: a generic framework including parallel and multiple-level designs

**DOI:** 10.1002/sim.6325

**Published:** 2014-10-24

**Authors:** Karla Hemming, Richard Lilford, Alan J Girling

**Affiliations:** Department of Public Health, Epidemiology and Biostatistics, University of BirminghamU.K.

**Keywords:** stepped-wedge, cluster, sample size, multiple levels of clustering

## Abstract

Stepped-wedge cluster randomised trials (SW-CRTs) are being used with increasing frequency in health service evaluation. Conventionally, these studies are cross-sectional in design with equally spaced steps, with an equal number of clusters randomised at each step and data collected at each and every step. Here we introduce several variations on this design and consider implications for power.

One modification we consider is the incomplete cross-sectional SW-CRT, where the number of clusters varies at each step or where at some steps, for example, implementation or transition periods, data are not collected. We show that the parallel CRT with staggered but balanced randomisation can be considered a special case of the incomplete SW-CRT. As too can the parallel CRT with baseline measures. And we extend these designs to allow for multiple layers of clustering, for example, wards within a hospital. Building on results for complete designs, power and detectable difference are derived using a Wald test and obtaining the variance–covariance matrix of the treatment effect assuming a generalised linear mixed model. These variations are illustrated by several real examples.

We recommend that whilst the impact of transition periods on power is likely to be small, where they are a feature of the design they should be incorporated. We also show examples in which the power of a SW-CRT increases as the intra-cluster correlation (ICC) increases and demonstrate that the impact of the ICC is likely to be smaller in a SW-CRT compared with a parallel CRT, especially where there are multiple levels of clustering. Finally, through this unified framework, the efficiency of the SW-CRT and the parallel CRT can be compared.

## 1. Introduction

Stepped-wedge cluster randomised trials (SW-CRTs), in which clusters are sequentially randomised until the point at which all clusters are exposed to the intervention, are being used with increasing frequency in the evaluation of service delivery interventions 1,2. Like parallel cluster randomised trials (CRTs), stepped-wedge cluster studies are used to avoid contamination or when the intervention needs to be delivered, or evaluated, at the population level 3–5. However, in addition, stepped-wedge studies are thought to be appropriate where there is already a belief that the intervention is expected to be of benefit and unlikely to do any harm, when evaluating a new service delivery intervention that will be implemented irrespective of evidence for effectiveness, or when it will be logistically implausible to roll out the intervention simultaneously to all clusters.

Power calculations are clearly established as important components in the design of any randomised study. In the early history of CRTs, power calculations were reported to be either incorrectly or inadequately performed, presumably because of the additional complication of clustering 6. With the onset of more applied research and increased methodological awareness of appropriate power calculations for CRTs 7, recent CRTs are more likely to be adequately powered and to contain an appropriate power calculation 8.

The literature on stepped-wedge studies is in its infancy. The earliest reports of published stepped-wedge studies are recent 1,2, and just 25 were identified in a recent systematic review 2. Furthermore, of those SW-CRTs identified in the earlier systematic review, only 8 out of 15 had a clear power calculation included 2. This suggests that, as was the case in the design of early CRTs, SW-CRTs might be inappropriately powered.

Stepped-wedge cluster designs are a form of cluster trial, and analysis and design needs to take account of this clustering. However, determination of sample size parameters (such as power and detectable difference) for stepped-wedge designs are not simple modifications of those for individually randomised designs 9. Whilst there is limited published work on power calculations in stepped-wedge studies, a theoretical formula for power has been established in the seminal paper by Hussey and Hughes 9 and a corresponding design effect established 10. However, the stepped-wedge designs considered by Hussey and Hughes, whilst a very useful addition to the literature, are for stepped-wedge designs where data are obtained or collected for the analysis at each and every step (we call this a complete design).

As applied health researchers, we have been involved in the design of several SW-CRTs with incomplete designs; that is, at some steps, and for some clusters, data are not collected or not intended to contribute to the analysis. Often these incomplete designs involve only the incompleteness brought about by an implementation phase; that is, a phase during which the cluster transitions from the control to intervention arm and so clusters during these periods can neither be considered to be a control nor fully exposed to the intervention 11. However, other designs have involved incompleteness other than implementation phases 12. To the extent that relaxing the typical features of the SW-CRT and allowing designs that are very incomplete, these designs can be viewed as including the conventional parallel CRT and the parallel CRT with baseline measures (sometimes called an analysis of covariance (ANCOVA) design in a cohort set-up), as a special case. We have also been involved in the design of SW-CRTs in which there exist multiple layers of clustering, for example, wards within a hospital.

In this paper, we introduce some modifications to the conventional SW-CRTs by considering incomplete designs and designs with multiple layers of clustering. We suggest a framework for analysis and from this derive a formula for estimating power. As a prelude, for completeness, we also summarise briefly the corresponding power formula for conventional cluster studies and SW-CRTs with complete design. Note that all of the studies considered here are cross-sectional in nature; that is, at each point in time it is assumed that the sample included in the study are different from those at other points in time.

## 2. Variations on stepped-wedge study designs

In this paper, we consider several extensions to the conventional SW-CRT and illustrate these by example in what follows. These are very pragmatic design variations that allow incorporation of design features that are likely to occur in practice and will have implications for power and sample size. These design variations are introduced here, and in the following sections we build on power formulae developed by others to incorporate the variations considered. By putting all of the related designs within a single framework, designers of studies will be much more able to see the impact of study design decisions on power and efficiency.

### 2.1. The conventional complete stepped-wedge cluster randomised trials

There are a number of defining characteristics that are typically features of the SW-CRT. These include a baseline collection period, where no clusters are exposed, and sequential random crossover to the intervention, which cannot be reversed for all clusters.

**Figure 1:**
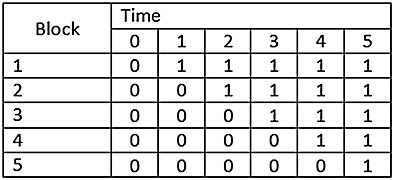
Illustration of a stepped-wedge study of complete design.

The conventional stepped-wedge design thus assumes that at each of a fixed number of points in time clusters will sequentially be randomised to the intervention and that at each point in time observations will be captured to form the data for the analysis. We refer to this as a complete design. We are limiting our consideration to cross-sectional designs, that is, designs in which the observations are collected on different participants at each step. We are therefore not concerned with cohort designs, in which participants are repeatedly followed up over time. Figure 1 illustrates a complete design with six time points and clusters randomised to blocks. It is conventionally assumed that the clusters within each block are independent and that the size of the blocks are the same. In Figure 1, cells with a “1” indicate that the clusters within that block at that point in time are exposed to the intervention; and cells with a “0” indicate that clusters within that block at that point in time are not exposed to the intervention. So, for example, there may be 20 clusters, with four randomised to cross from control to intervention at each of five steps and with a single period of baseline data collection. We call this representation of the design of the study a “design pattern matrix”.

### 2.2. The incomplete stepped-wedge cluster randomised trial

**Figure 2:**
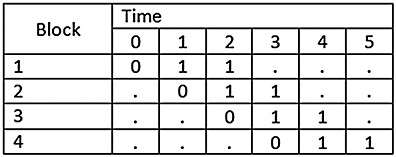
Illustration of a stepped-wedge study of incomplete design with one before and two after measurements.

**Figure 3:**
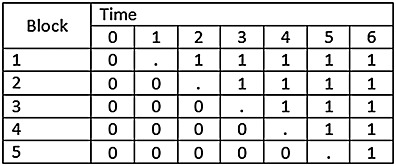
Illustration of a stepped-wedge study of incomplete design with an implementation period.

In practice, designs may not be so complete as those described previously. Figures 2 and 3 illustrate what we have called the incomplete design. In the incomplete design, a cluster, or block of clusters, may be either exposed to the intervention (denoted by “1" ), not be exposed to the intervention (denoted by “0" ) or not contribute to the analysis (denoted by “·" ). Figure 2, for example, illustrates a design in which clusters are randomised sequentially, but for each cluster only baseline observations and two follow-up observations are made. In Figure 3, we illustrate a design in which data are collected throughout the study period, except for the time in which the intervention is being implemented, sometimes called a transition or implementation phase. An implementation phase represents a period in which the cluster transitions from the control to intervention arm, and so clusters during these periods can neither be considered to be a control nor fully exposed to the intervention.

### 2.3. The staggered parallel cluster randomised trials with baseline measures

**Figure 4:**
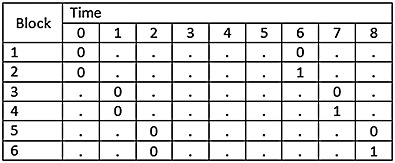
Illustrative example of staggered, but parallel, CRT.

One of the often cited reasons for conducting a SW-CRT is that it is impractical to roll out the intervention in a large number of clusters simultaneously. An alternative to the SW-CRT is to conduct a variation of the parallel CRT in which the roll-out (and thus randomisation) is staggered, but crucially the design is balanced on time. An illustration of such a design is provided in Figure 4.

In this parallel, but staggered, CRT, at three points in time, a number of clusters are randomised to the intervention or control arm, with baseline and follow-up measures taken. Parallel CRTs with baseline measures are sometimes referred to as ANCOVA designs, when they involve repeated measures on the same individuals 13. However, the design considered is under the set-up where there are different individuals at baseline and follow-up. This design is a parallel CRT, staggered over time but with balanced allocation to intervention and control arms, with a single before and single after measurement. An alternative design would be one without baseline measures, so just a simple parallel CRT.

This design pattern does not include the typical features of a SW-CRT, as not all of the clusters cross over to the intervention. However, this design (and likewise the simple parallel CRT) might also be visualised using a design pattern matrix. We go on to show that, using the design pattern matrix as a schematic representation of the study, parallel CRTs (with or without baseline data collection) can be viewed within the same framework as the SW-CRT.

### 2.4. The stepped-wedge cluster randomised trial with multiple layers of clustering

**Figure 5:**
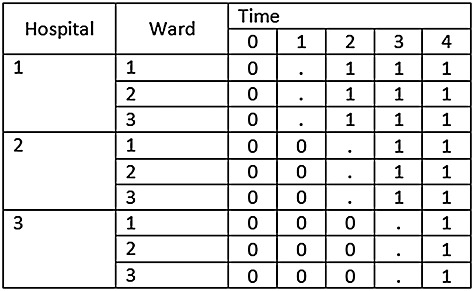
Illustrative example of SW-CRT with two layers of clustering (wards within a hospital).

Cluster trials are sometimes conducted in settings with multiple layers of clustering 14. For example, a CRT may be conducted across multiple hospitals and multiple wards within each hospital, or clusters may represent geographical regions with several hospitals within each region, or classes may be grouped within schools in studies investigating effectiveness of interventions in school settings. It is therefore plausible that multiple layers of clustering may also occur within stepped-wedge studies. Such a design is illustrated in Figure 5. In this example, there are three hospitals and three wards in each hospital. The order in which the hospitals cross over to the intervention is randomised, with all wards within each hospital crossing over at the same point in time (to avoid contamination). Other than this, the design follows a conventional SW-CRT design with the addition of a transition period.

## 3. Background

### 3.1. Power for parallel cluster randomised trials

In a typical randomised controlled trial (RCT), the intervention effect can be expressed as the mean difference in outcome between the two arms of the trial. The statistical significance of such a difference can be assessed using a test statistic of the form 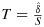 where 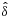 is the average difference in outcome as observed in the trial and *S* is a sample estimate of its standard deviation. For large trials, *T* will follow a normal distribution with unit standard deviation, at least approximately, and the difference can be declared significant at the 100*α%* level provided that

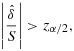
1
where *z*_*α*_/2 is the 100*α* upper percentage point of the standard normal distribution (=1.96 for tests at the 5*%* level). In practice, this procedure may not be optimal unless the trial is a large one, but it does support a simple and effective approach to power and sample size calculations. In this approach, sampling fluctuations in the estimate *S* are ignored, and *S* is replaced by its exact theoretical value, fully justifiable if the trial is large.

#### 3.1.1. Randomised controlled trials with equal-sized arms

One common situation arises when 2*n* individuals are randomised equally between two treatment arms. In this case, *S* approximates to 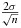 where *σ* is the standard deviation of an individual patient outcome, assumed to be the same in each arm. If the two arms have different standard deviations (which may arise, for example, if the outcome is binary), *σ* will represent a pooled standard deviation. In the binary case,

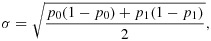
2
where *p*_0_ and *p*_1_ are the binary probabilities in the two arms of the trial. In any case, the statistic *T* approximates to 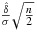, and the result is declared significant if

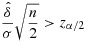
3
or

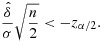
4
The power of the procedure (replacing 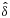 with *δ* the treatment effect to be detected) is just the sum of the probabilities of these two events. Now suppose that the true intervention effect is positive, i.e. that *δ* > 0. Then, in a large trial, it is unlikely that the second event will happen and so almost all the power derives from the first possibility. (The situation is reversed when *δ* < 0, in which case the first possibility can be disregarded). This leads to a standard approximation for the power, given by (1 − *β*) where *β* is derived from the following equation:

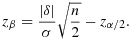
5
This formula can be rearranged to determine the absolute value of the minimum detectable difference (*δ*_*min*_) for fixed power and sample size:

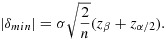
6
In a CRT the same formulae can be used, but the variance of the outcome (*σ*^2^) must first be inflated by a design effect (DE). For clusters of equal size (*m*), the DE is given by the following:

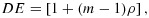
7
where *ρ* is the ICC defined as the correlation between the outcomes for two individuals in the same cluster or, equivalently, as the proportion of the variance attributable to variation between clusters. The corresponding power formula is as follows:

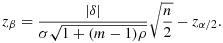
8
Note that if the outcome variance is given by 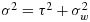, where *τ*^2^ is the variance of the cluster means and 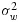 is the variance within clusters, the ICC can be expressed as 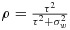.

## 4. Stepped wedge designs

### 4.1. Power for complete stepped-wedge designs

In a stepped-wedge design, each cluster receives the intervention eventually, but the clusters are randomised to receive it at different times. In a complete cross-sectional design, the time at which the intervention is received is different for each cluster, and (independent) observations are taken in every cluster at all time epochs from the start of the study, when no clusters have received the intervention, until the end when the last cluster to be randomised has received the intervention. In this design, the number of time epochs is one more than the number of clusters. A model for this situation has been proposed by Hussey and Hughes 9:

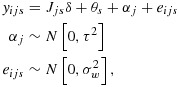
9
where *i* indexes the individual (*i* = 1,…,*m*), *j* indexes the cluster (*j* = 1,…,*k*) and *s* indexes the time points (*s* = 1,…,*k* + 1), with fixed effects for time (*θ*_*s*_) and random cluster effects (*α*_*j*_). Here *y*_*ijs*_ is the outcome, *δ* is the intervention effect and *J*_*js*_ is a binary variable that takes the value 1 if cluster *j* has been exposed to the intervention by time *s*. It is assumed that there are exactly *m* observations in each cell of the design, that is, for each time by cluster combination. This is a slight variation in our earlier notation because the number of observations per cluster over time is now *m*(*k* + 1) and not *m* as in the earlier paragraphs. Of note, this model is for a cross-sectional design only.

Hussey and Hughes propose to determine the power of this design by considering a Wald test for the intervention effect *δ*. If the variance components (*τ*^2^ and 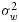) are known (as is generally assumed in power calculations), it is sufficient to consider the following model for the cell means:

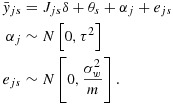
10
The *k*(*k* + 1) by *k*(*k* + 1) variance–covariance matrix (*V*) of the cell means (ordered by time within cluster) has a block-diagonal form with *k* identical (*k* + 1) by (*k* + 1) matrices (*V*_*j*_) on the diagonal, each representing a single cluster. The zero off-diagonal matrices reflect the fact that different clusters are independent of one another; that is,

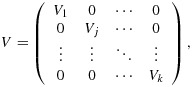

where each block *V*_*j*_ (of size *k* + 1 by *k* + 1) is of the following form:

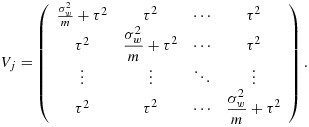

In this linear mixed model, there are *k* + 2 unknown linear parameters: the intervention effect, *δ*, and the time parameters *θ*_1_,…,*θ*_*s*_ (where *s* = *k* + 1). The variance–covariance matrix for their estimated values takes the form (*X*^′^*V*^−1^*X*)^−1^, where *X* is the *k*(*k* + 1) by (*k* + 2) design matrix that describes the model for the cell means. For this complete stepped-wedge design, the power formula is as follows:

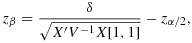
11
where the notation [1,1] refers to the matrix cell in the first column and first row (assuming that the design matrix is ordered by treatment indicator (*J*) and then time indicators).

### 4.2. Power for complete stepped-wedge designs with multiple layers of clustering

This approach can be extended to accommodate non-independent grouping within the clusters in a stepped-wedge study. Let *g* represent the number of groups within each cluster. For example, we might imagine that a SW-CRT will be conducted across *k* hospitals with *g* wards in each hospital. To accommodate this extra level of variation, an additional variance component can be introduced into the mixed effects model so that

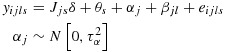
12

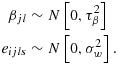
13
Here *α*_*j*_ stands for the *j*th cluster (hospital) effect and *β*_*jl*_ is an effect for the *l*th group (ward) within the *j*th cluster, and again we assume a cross-sectional design. For power calculations, the variance components are assumed known, and it is again sufficient to consider the model for the mean observation in each cell of the design, that is, ward by group by time combination. Here there are *k*(*k* + 1)*g* cells. If the cells are ordered by time within groups within clusters, the variance–covariance matrix (*V*) for the cell means is again block diagonal, with identical diagonal blocks (*V*_*j*_). In fact,

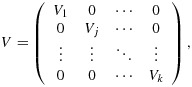

with *V*_*j*_ a *g*(*k* + 1) by *g*(*k* + 1) matrix of the form

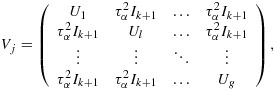

where *I*_*k* + 1_ is the identity matrix of order *k* + 1. The (*k* + 1) by (*k* + 1) matrix *U*_*l*_ refers to the variation of cell means over time within the same group (ward) so that

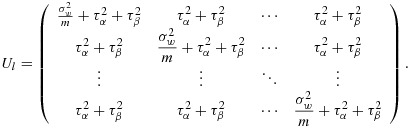

The correlation structure in a study with a single layer of clustering is characterised by the ICC coefficient. This may be defined either as the correlation between two observations in the same cluster or, equivalently, as the proportion of the individual variance attributable to cluster membership. In a study with a single layer of clustering, the individual variance is written as 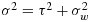, where *τ*^2^ is the variance of the cluster means and 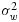 is the within-cluster variance (i.e. the conditional variance of an observation given the cluster to which the individual belongs). Then the ICC is

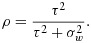
14
In a study with two layers of clustering, we might think of having two ICCs. These might be paramaterised in one of several ways 15. Here, we define the first ICC as the correlation between two groups within the same cluster (i.e. between two wards within the same hospital) and the second ICC as the correlation between two observations within the same group (i.e. correlation between two observations within the same ward). So the first ICC here is

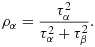
15
And the second ICC is

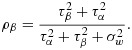
16
The total variation is now partitioned into within cluster, between group and between cluster:

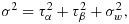
17
so that

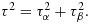
18
We also note, however, that in parallel CRTs with multiple layers of clustering, a conservative approach to estimate the power is to treat the groups within clusters as one large cluster 16. For example, in a parallel CRT with clusters of hospitals and wards within hospitals, treating the hospitals as one large cluster will provide a conservative estimate of power. Through an example, we investigate whether this is also the case for SW-CRTs.

### 4.3. Power for incomplete stepped-wedge designs

The aforementioned formula can be modified for incomplete designs, simply by taking the appropriate design matrix for the incomplete design and the appropriate block-diagonal matrix *V*. *V* will remain a block-diagonal matrix and will contain the same number of blocks as clusters, but this time each block will be of varying size depending on the number of observation time points taken for cluster *j*. At this point we generalise the design to allow for *s* time points (in the complete design *s* = *k* + 1). So, for example, if in one cluster there are no observations taken at one point in time, the size of the block matrix *V*_*j*_ will be *s* − 1 by *s* − 1 rather than *s* by *s*. For example, for the very simple design of two clusters (*k* = 2) and four time points (*s* = 4), with an implementation period, the design pattern matrix is as follows:

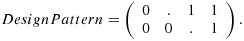

Simplifying notation temporarily and letting *t* represent time (there are four time points), *c* represent the cluster (there are two clusters), and *J* represent exposure to the intervention, the corresponding design matrix is as follows:

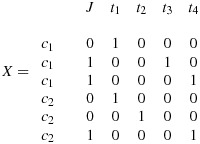

*V* remains a block-diagonal matrix, with one block per cluster,

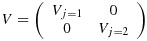

and where *V*_*j*_ (*j* = 1,2) is now of the dimension *s* − 1=3 by *s* − 1=3 and is of the form

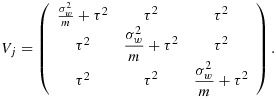


### 4.4. Power for staggered parallel cluster randomised trials

When visualising the design of a study by its design pattern matrix, many study designs can be considered as a special case of this more general SW-CRT design. This includes the parallel CRT, the parallel (cross-sectional) CRT with baseline measures (commonly called the ANCOVA design when the same individuals are measured at baseline and follow-up 13). And, as a special case, the parallel, staggered but balanced, CRT (Figure 4). This means that the formula provided previously can be used to estimate power under these designs, which may not conventionally be thought of as SW-CRTs.

However, others have shown that an alternative way of establishing power in the parallel CRT with baseline measures is to consider the design as a conventional parallel CRT, adjusting for baseline measures 13. Using their approach, the implications that adjustment for baseline measurements have on sample size calculations can be formulated by estimating the correlation (*r*) between the baseline measurements and the outcome (that is, between cluster means) and reducing the sample size by a factor *r*^2^. For a given ICC, *ρ*, it can be shown that the correlation between cluster means, *r*, is as follows:

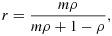
19
where *m* is the cell size (so there will be 2*m* measurements per cluster).

Under a cross-sectional design, this means the total sample size will be inflated by that under individual randomisation by 2*[1 + (*m* − 1)*ρ*](1 − *r*^2^) (using *m* as the cell size and multiplied by 2 to count the pre-measurement and post-measurement). We compare this with the DE that would be obtained in the parallel CRT, with 2*m* measurements per cluster (i.e. [1 + (2*m* − 1)*ρ*]).

**Figure 6:**
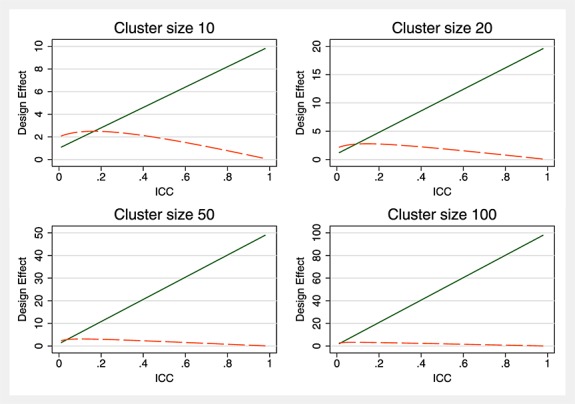
Design effects for parallel CRT (solid line) and before and after (dashed line) designs.

In Figure 6, we show the ratio of sample sizes required under a parallel CRT design and a parallel CRT design with baseline measurements, both compared with the sample size required under individual randomisation (i.e. design effects) for a range of cluster sizes. Whilst the design effect for a parallel CRT has a clear linear relationship with the ICC, this is not the case for the design effect for a parallel CRT with baseline measures.

This means that when planning a parallel CRT with baseline measures, power can either be estimated by assuming that the trial is an incomplete SW-CRT or by using conventional power methods for a parallel CRT and using the estimated ICC to determine the adjustment factor, *r*, for baseline measures. Both methods lead to identical estimates of power.

## 5. Examples

We illustrate these novel concepts by three examples. In the first example, a service delivery intervention applied to the entire cluster, there is (as is perhaps more common) a single layer of clustering but an incomplete design both to allow for an implementation phase and pragmatic limitations in the design due to data collection limitations. In the second example (again service delivery and applied to the entire cluster), there is a complete design pattern, but there is an additional layer of clustering (the unit of randomisation is region, but within each region there are several hospitals). In the third example, the intervention cannot be simultaneously delivered to all clusters, although it is not required to deliver the intervention to all clusters, and so a staggered parallel CRT is considered.

All of the formulae necessary for the power calculations conducted were provided earlier. These formulae, apart from the two-level extension, have been implemented in two Stata functions, one *clustersampsi*, a sample size calculator for CRTs 17, and the other Stata programme, *steppedwedge*, a power calculator for SW-CRTs 18. An extension of *steppedwedge* for two levels of clustering (i.e. wards within hospitals) is available from the authors on request.

### 5.1. Example 1: a stepped-wedge cluster randomised trial with an implementation phase

A stepped-wedge design is to be used to evaluate whether the provision of a training scheme improves the rates at which midwives perform membrane sweeping in post-term pregnancies. Membrane sweeping is a simple and inexpensive procedure and has been shown to reduce the need for formal induction of labour. The training scheme is to be implemented over a period of time by training midwives in groups of community teams. The training scheme will be implemented irrespective of evidence of effectiveness, but the two trusts involved have agreed to evaluate the intervention using a stepped-wedge design.

**Figure 7:**
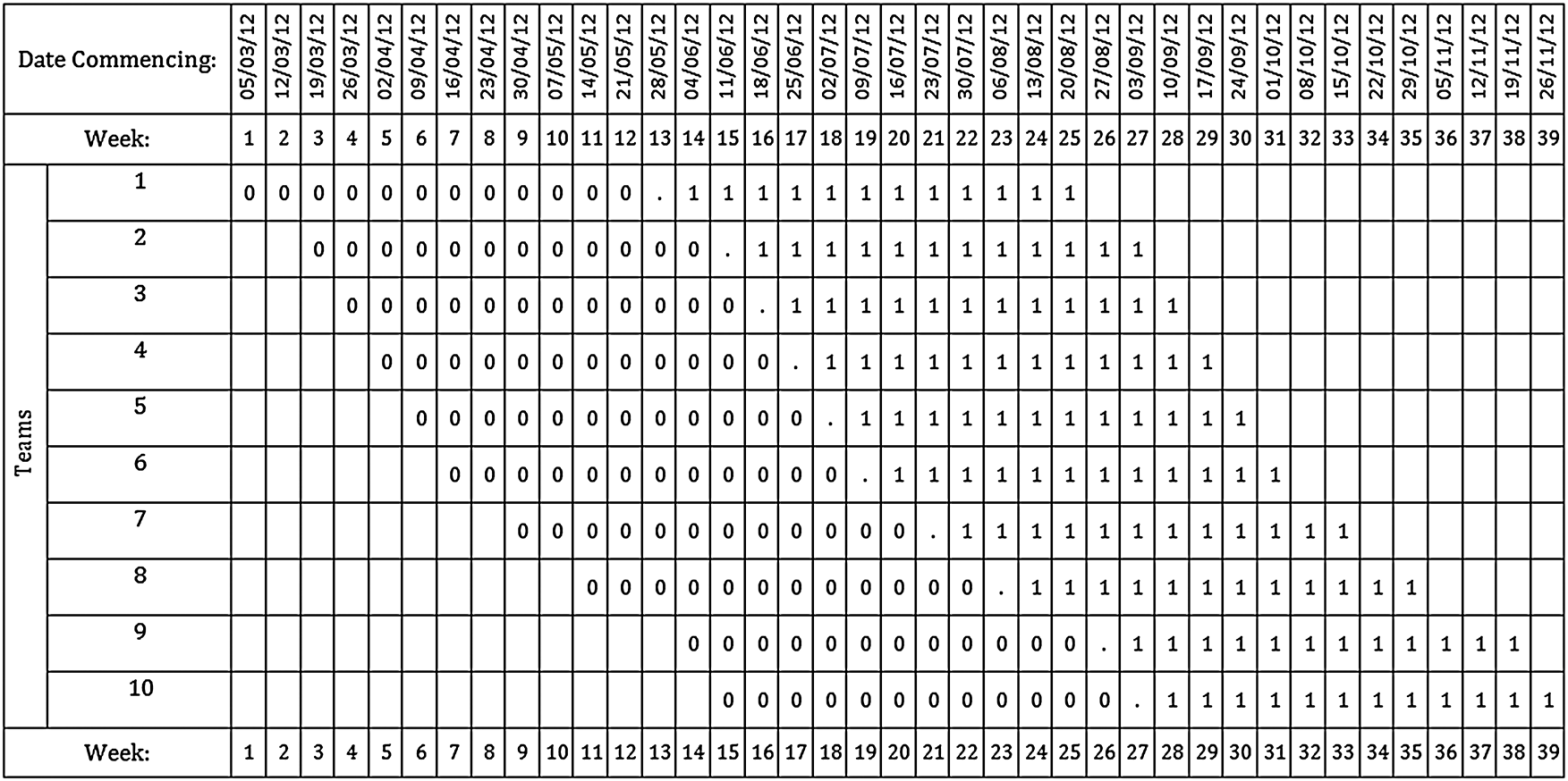
The sweeping study, an illustrative example of stepped-wedge study of incomplete design (Example 1).

There are 10 community teams and so 10 clusters, with approximately 12 births occurring within each team each week. Teams will be randomised to the order in which they receive the intervention in a design that includes, for each cluster, a 12-week period of pre-implementation observational data, a week transition period (during which the training will be delivered) and a 12-week period of post-implementation data. In addition, to allow for holiday periods and other pragmatic reasons, whilst the roll-out will usually be staggered by 1-week periods, these periods will sometimes be longer (2 or 3weeks). This uneven nature of the design has been built into the design pattern matrix. So for example, between randomisation of clusters 8 and 9 rather than a 1-week step, the step is of length 2weeks to accommodate a known holiday period during which no training will be delivered (Figure 7). The design is therefore an incomplete stepped-wedge design. It has been postulated that a clinically important difference to detect in the proportion of women having a membrane sweep is between 40*%* and 50*%*. There is little literature to guide likely values of the ICC, and so we investigated a range of values but present results here for a single value only 19. For a two-sided test and 5*%* significance level and assuming an ICC of 0.01, using the design pattern matrix as illustrated in Figure 7, with 12 observations per cell (that is 12 women giving birth per team per week) equating to a total of approximately 2880 observations, we compute that there would be in the region of 78*%* power. Ignoring the implementation phases (assuming half of the clusters were unexposed and half exposed) results in a slightly over-optimistic estimate of the power at about 84*%*.

### 5.2. Example 2: a stepped-wedge cluster randomised trial with multiple levels of clustering

The Enhanced Peri-Operative Care for High-risk patients (EPOCH) study proposes to conduct an evaluation of a series of educational interventions designed to reduce mortality in patients undergoing emergency laparotomy. Part of the intervention involves group training, and so for pragmatic reasons the intervention will be rolled out so that hospitals that are in the same region initiate the intervention at the same point in time. That is, region will be the unit of randomisation, but within each region there is clustering due to hospitals. Here we determine whether the trial would be more or less efficient (in terms of total number of measurements required) under a stepped-wedge design and also whether the stepped-wedge design would be more or less sensitive to the value of the ICC.

In parallel CRTs with multiple layers of clustering, a conservative approach to estimate the power is to treat the clusters within clusters as one large cluster. Through an example, we demonstrate this here. We then investigate the impact of this assumption for SW-CRTs. In this example, *ρ*_*α*_ represents the correlation between hospitals within the same region and *ρ*_*β*_ represents the correlation between two observations within the same hospital. As the unit of randomisation is the region, we consider sensitivity to three values for *ρ*_*β*_ (ignoring the clustering within clustering, *ρ*_*β*_ would be the conventional ICC). For *ρ*_*α*_ we consider sensitivity across values 0 to 1. Fixing *ρ*_*α*_ at 0 is equivalent to assuming that two hospitals within the same region are completely independent (optimistic), and fixing *ρ*_*α*_ at 1 is equivalent to assuming that two hospitals within the same region are one entity (conservative).

It is anticipated that 96 hospitals will participate in the study, grouped into 16 regions (*k* = 16 clusters) with each region containing six hospitals (*g* = 6 groups). The study will run over 17 time periods where each period of time is approximately 1month. It is expected that there will be approximately 18 emergency laparotomies per month in each hospital (*m* = 18). The primary outcome is mortality, and current mortality rates are estimated to be about 10*%*. Clinically important reductions in risk of death are postulated to be about a 2*%* absolute reduction. It is expected that there will be correlation within hospitals and also within groups of hospitals (regions). We illustrate this example across a range of correlations.

This study might be conducted as a parallel CRT with an estimated group (hospital) size of 306 = (18*17) and cluster size (six hospitals) of 1836 = (306*6), giving a total sample size of 29,376 = (1836*16). Alternatively, the study might be conducted as a SW-CRT. Under a SW-CRT design, it is anticipated that there will be a baseline phase (lasting 1month), in which no hospitals are exposed to the intervention, and one period at the end of the study in which all hospitals will be exposed to the intervention. This is a complete design with no transition periods, with 16 steps and 16 clusters. The cell size for each group (hospital) would be 18, and the cell size for each cluster (region) would be (108 = 18*6). Again, the total sample size for this design would be 29,376(=16*17*108).

**Figure 8:**
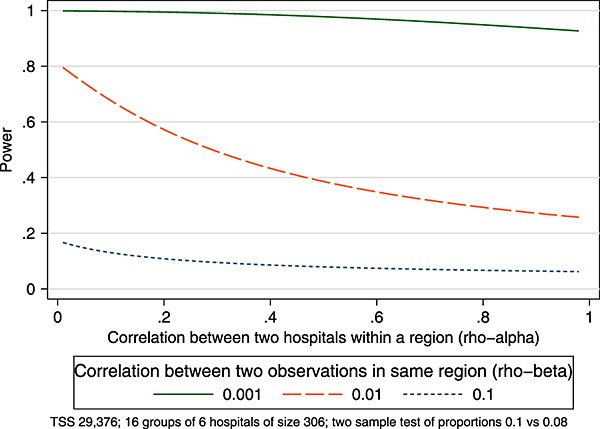
Influence of clustering within clustering in a parallel CRT.

Consider first this example under the parallel CRT design with eight clusters randomised to the intervention and eight to the control, and each of these clusters consists of six groups, each cluster of size 1836. Suppose initially we ignore the grouping within the clusters and assume that there is a single level of clustering only; that is, there are eight clusters in each arm, each of size 1836. We then compare this with the power we obtain by correctly acknowledging the two levels, that is, that there are eight clusters, with six groups within each cluster, each of size 306. Figure 8 illustrates that for this example, a parallel CRT, assuming there is just one large cluster (setting *ρ*_*α*_=1) provides a conservative estimate of power over the range of ICCs (*ρ*_*β*_) compared with the correct method of acknowledging the clustering within clustering (*ρ*_*α*_<1). However, whilst conservative, this estimate can be very un-optimistic or highly sensitive on the value *ρ*_*α*_ (the extent of the correlation between hospitals within the region).

**Figure 9:**
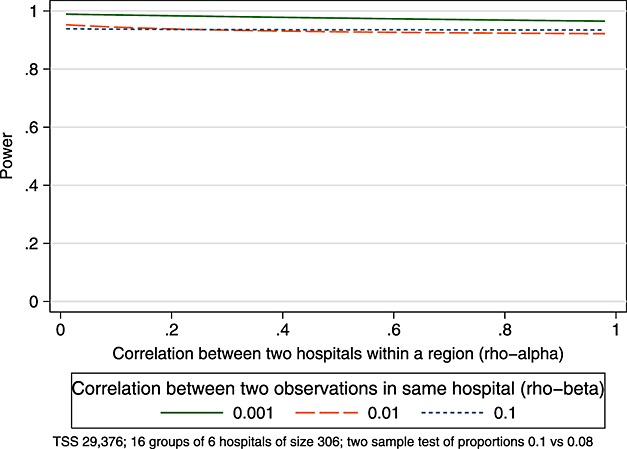
Influence of clustering within clustering in a SW-CRT.

Now, suppose instead of a parallel CRT we have a complete SW-CRT with 16 steps, 16 clusters within which there are six groups. We first estimate power, as discussed previously, ignoring this grouping and treating each group of six hospitals as one large cluster (cell size 108); that is, we assume a single level of clustering (*ρ*_*α*_=1). Secondly, we rightly assume that each cluster is composed of six groups, each of cell size 18. Figure 9 shows how power varies by *ρ*_*α*_ and *ρ*_*β*_. Unlike in the parallel CRT example discussed previously, assuming a single level of clustering in a SW-CRT is not highly sensitive to the extent of the correlation between hospitals within the region (*ρ*_*α*_).

This example also illustrates that the power under the CRT quickly diminishes as the ICCs increase, whereas under the SW-CRT the power is less sensitive to the increase in ICC, a point to which we return to in the next example. For this study, it is therefore the case that under a stepped-wedge design, the power will be less sensitive to any mis-specification of the ICC, less sensitive to any mis-specification of the two-level nesting structure (hospitals within regions) and more powerful (for the same sample size) than the parallel cluster design.

### 5.3. Example 3: a staggered parallel cluster randomised trial

A staggered parallel CRT is the proposed design to evaluate the effectiveness of a multifaceted nutritional intervention programme delivered through early years care settings, in children aged 2 to 3 years. It is anticipated that funding will allow the intervention to be delivered across nine centres and nine control centres (*k* = 18), each of size approximately 15 (*m* = 15). A sample of children will be measured at baseline and another sample measured at follow-up, so that a total of 540 observations will be taken.

**Figure 10:**
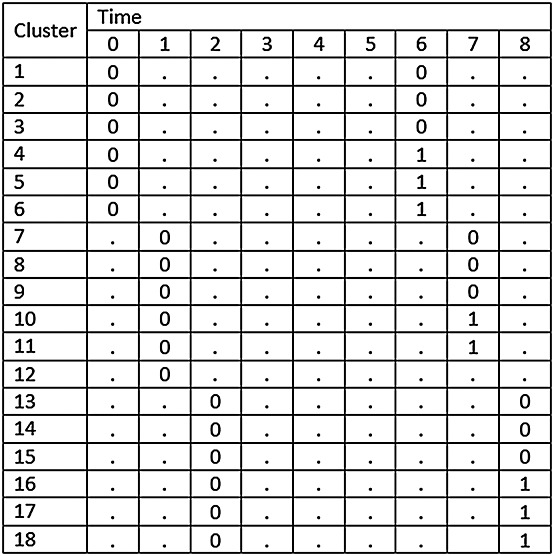
The nursery study, an illustrative example of a staggered but parallel cluster trial (Example 3)

It is anticipated that the intervention can be rolled out in three centres simultaneously. Therefore, centres will be recruited and randomised in blocks of six (three control and three intervention). Baseline measures will be collected in all clusters. The design is illustrated in Figure 10.

The main outcome is number of fruit and vegetable portions eaten per day, and it is hoped that the intervention will lead to an increase intake of about one portion (SD of 2.2). There is a dearth of information on likely values of the ICC for this outcome, although for process outcomes ICCs are typically higher, and so we consider a range between 0.05 and 0.5. For each ICC, using Equation 28, we also provide the estimated correlation between the cluster means for the cell size of 15 (m).

Power may be computed assuming that the design is an incomplete SW study or equivalently by assuming that the design is a parallel but staggered CRT with baseline measure. Under the parallel CRT with baseline and follow-up measures, power calculations require an estimate of the correlation between baseline and follow-up cluster means (derived from the ICC using formula in Equation 28).

Table 1 shows that whilst power initially decreases with increasing ICC, perhaps un-intuitively, the power then begins to increase with increasing ICC. This is consistent with the non-linear relationship seen in Figure 6 between the design effects and the ICC.

**Table 1:** Parallel CRT with baseline measures.

ICC	Correlation	Power
0.05	0.44	0.891
0.1	0.63	0.870
0.15	0.73	0.869
0.2	0.79	0.877
0.3	0.87	0.905
0.4	0.91	0.937
0.5	0.94	0.967

## 6. Discussion

We have provided a unified framework for the design of CRTs, with or without baseline measures and staggered or fixed randomisation in time. This unified framework includes as special cases the conventional parallel CRT, the parallel CRT with baseline measures (known as the ANCOVA design when the same individuals are measured at baseline and follow-up), staggered over time parallel CRTs and the conventional SW-CRT in which all clusters are randomised sequentially to cross from the control to intervention arm. However, in addition to this, this framework also includes designs that are less conventional, for example, the SW-CRT with transition periods. These models also include extensions to two levels of clustering, for example, wards within a hospital. Such a unified framework will allow more straightforward comparisons of efficiency of the various designs.

Our examples have demonstrated that, unlike the conventional parallel CRT, power does not have a linear relationship with the ICC. For small values of ICC, we observed the power to decrease with increasing ICC. But for larger values of the ICC, power increased with increasing values of the ICC. Furthermore, this result also holds true for the conventional parallel CRT with baseline and follow-up measures. That is to say that in a conventional parallel CRT with baseline and follow-up values, above some critical value power will actually increase with increasing ICC, a result that we do not believe is widely appreciated but is probably explained by the relationship the ICC has with the correlation between measurements.

In a CRT with multiple layers of clustering, illustrative examples here support the hypothesis that treating the groups of clusters as one large cluster will result in a conservative estimate of power. However, whilst conservative, this assumption is highly sensitive to the extent of the correlation between the clusters within the cluster (i.e. wards within a hospital). However, in the example SW-CRT considered here, this is not the case, and treating the groups within a cluster as one big cluster whilst also conservative is not very dependent on the extent of the correlation between groups within the same cluster. Whilst we have not formally proven this relationship, this is an intuitive finding, as SW-CRTs are known to be less sensitive to the ICC than parallel CRTs, and so it is natural that the SW-CRT should be less sensitive to ignoring any additional hierarchical structure within the design.

In the conventional SW-CRT, observations (i.e. data) are gathered from every cluster at each and every step within the study. Any analysis using data from each and every step must consequently assume that the cluster was either exposed to the intervention or was not exposed at each point in time. However, in many intervention studies there will be a period of bedding in, during which the intervention becomes embedded in practice, and during this transition period the cluster can neither be considered exposed to the intervention nor not exposed. If data from such transition periods are not included in the formative evaluation, then neither should they be counted in the power calculation. The formula presented here, and examples, will allow designers of SW-CRTs to properly allow and build in transition periods into SW-CRT study designs.

It is important to note that the formulae and examples considered here apply to cross-sectional designs only. There are several important aspects to consider when extending the SW-CRT from the cross-sectional design to the cohort design. Firstly, powering such studies will necessitate the incorporation of the dependence between repeated measures on the same participants. Secondly, individual patient recruitment becomes less appealing in SW-CRTs, because of the intensity of data collection (outcomes must be measured at each and every point in time). And finally, whilst CRTs are an accepted method of study design in situations where individual participants need to be recruited, it has also been demonstrated that such designs can suffer from selection biases where recruitment of individuals takes place after the allocation is known 20,21. Given that in SW-CRTs, with individual patient recruitment, concealment of allocation is not possible (as in the case of CRTs), it is plausible that these selection biases might also be prominent in SW-CRTs.

Whilst we did not consider extensions to cohort designs, the two-level models considered here, for example, allowing for clustering within hospitals and within wards, could potentially be applied to repeated measures on the same individuals. In such cases, our two parameterisations for the ICCs would represent the correlation between two individuals within the same cluster (*ρ*_*α*_) and the correlation between two measurements in the same cluster (*ρ*_*β*_).

There is a dearth of literature on the theoretical and practical aspects of designing and analysing SW-CRTs. When designing such studies two important and necessary considerations are power and the potential for bias. We have extended power calculations for variations of the conventional SW-CRT, considered the alternative of the staggered CRT, and extended the SW-CRT for an additional layer of clustering. An important limitation of our work is the assumption of cross-sectional designs, but our two-level extension should be able to accommodate designs in which participants are followed up over time. Other potential limitations include lack of small sample correction, although we believe that designers should be encouraged to not carry out studies with small numbers of clusters. Other issues to consider are the impact of known variance assumptions in these calculations, which could be investigated through simulation studies.

There are many other important aspects that remain to be considered. There is much debate over whether the SW-CRT is an efficient and unbiased method of evaluation. Some have argued strongly against the use of the SW-CRT 22, whilst others have argued that SW-CRTs might have a valuable place in the hierarchy of evidence without which many interventions in service delivery might not be evaluated 23–25. There is also much debate over whether the SW-CRT is more efficient than the parallel CRT 10,26–31. Some have argued that it is more efficient 10, whilst others have argued that this will depend on the ICC 17. More research is urgently needed to resolve these issues.

## 7. Author contributions

A. Girling and K. Hemming conceived the idea. K. Hemming wrote the first and subsequent drafts. All authors read and approved the final manuscript.

## 8. Competing interests

The authors declare that they have no competing interests.
